# Pidotimod: the state of art

**DOI:** 10.1186/s12948-015-0012-1

**Published:** 2015-05-21

**Authors:** Beatrice E Ferrario, Silvia Garuti, Fulvio Braido, Giorgio W Canonica

**Affiliations:** Respiratory Diseases and Allergy Clinic, DIMI-Dept.Internal Medicine, University of Genova, IRCCS AOU S.Martino-IST, Genoa, Italy

**Keywords:** Pidotimod, natural immunity

## Abstract

Despite the use of antibiotics and vaccines, the frequency of respiratory tract infections is still high and these infections interest a wide range of patients, from children to aged people, including in particular these extreme categories because of the deficiency of their immune system, due to immaturity in the former case and to “immunosenescence” in the latter. For that reason immunostimulant drugs are getting more important to prevent and to attenuate infections. Pidotimod (3-L-pyroglutamyl-L-thiazolidine-4carboxylic acid) is a synthetic dipeptide with immunomodulatory properties. We reviewed studies conducted on different categories of patients, with particular attention on children and senile patients suffering from recurrent respiratory tract infections, associated, or not, with asthma or COPD. The outcomes considered are both clinical and laboratory parameters. The common end-point of these studies is that Pidotimod has an immunomodulatory activity which is able both to improve the clinical conditions of patients and to enhance and stimulate their immunity cells (lymphocytes but not only) functions acting on adaptive and innate immunity. Pidotimod is also able to increase the concentration of salivary IgA directed against bacteria; furthermore, it can modulate airway epithelial cells functions up-regulating the expression of toll-like receptors and acting on adhesion molecules. According to studies conducted on patients with atopic asthma, it seems that Pidotimod could affect T-lymphocytes balance with a possible addictional anti-allergic activity. Furthermore, it has been demonstrated an improvement of FEV1 and PEF in asthmatic patients treated with Pidotimod. Main clinical outcomes are the reduction of the number of infectious episodes, lesser severity of signs and symptoms and, consequently, a reduction in use of antibiotics and symptomatic drugs, less working and school days lost, less mortality and morbidity. The studies considered give positive results, confirming Pidotimod’s efficacy. Furthermore, many studies show a good safety profile of the drug, without recording serious adverse events and mutagenic potential, and a very low incidence of side effects. Pidotimod is also a more safe solution in patients subjected to vaccination, if compared to lyophilized polibacterial, which can’t be administered for thirty days before vaccination.

## Introduction

Acute respiratory tract infections (ARTIs) were called by some authors, and still are, the “forgotten pandemics”. At present, despite the introduction of new antibiotics and vaccines which contribute to reduce the risk of mortality and morbidity, they remain widespread and affect both young and elder people. Furthermore, the socio-economic burden of ARTIs remains high, considering the cost of symptomatic drugs, antibiotics, hospitalization and the indirect cost of absence from work or loss of school days.

In particular, viruses are the main agents responsible of ARTIs in the pediatric age but the high number of circulating viruses and their different sub-types, besides the environmental risk factors, are not the only factors contributing to the incidence of ARTIs in the first years of life. In fact, the immaturity of the immune system in children maintains a considerable role. That’s why a functional approach to preventing and treating ARTIs is increasing the immune response or enhancing the child’s innate defence mechanisms [1].

Also in elder people there is a deficiency in the function of immune system, which leads to frequent infections, in particular of respiratory tract, and to COPD (chronic obstructive pulmonary disease) exacerbations. The immune system, in fact, during senile age goes through age-associated alterations, defined as “immunosenescence” and still matter of study, which lead to a lower ability to respond to infections and to develop immunity after vaccination [2].

Since the early 90’s a wide scientific production has been published about Pidotimod, a new immunostimulant drug. Its efficacy has been tested both in children and in adults, with a focus of interest on respiratory tract infections, in particular as a complication of asthma and chronic obstructive pulmonary disease.

## Review

### The immunomodulating activity of Pidotimod

In the last years different kinds of immunostimulants of natural or synthetic origins and working with different mechanisms have been created for the prevention of ARTIs. Pidotimod (3-L-pyroglutamyl-L-thiaziolidine-4 carboxylic acid) is a synthetic dipeptide molecule with immunomodulatory properties [1].

In vivo and in vitro studies shows that Pidotimod’s immunomodulatory activity is focused on both adaptive and innate immunity. Pidotimod induces dendritic cells (DCs) maturation, up-regulates the expression of HLA-DR and of co-stimulatory molecules, stimulates DCs to release pro-inflammatory molecules driving T-cells proliferation and differentiation towards a Th1 phenotype, enhances natural killer (NK) cells functions and promotes phagocytosis [3].

**Figure 1:**
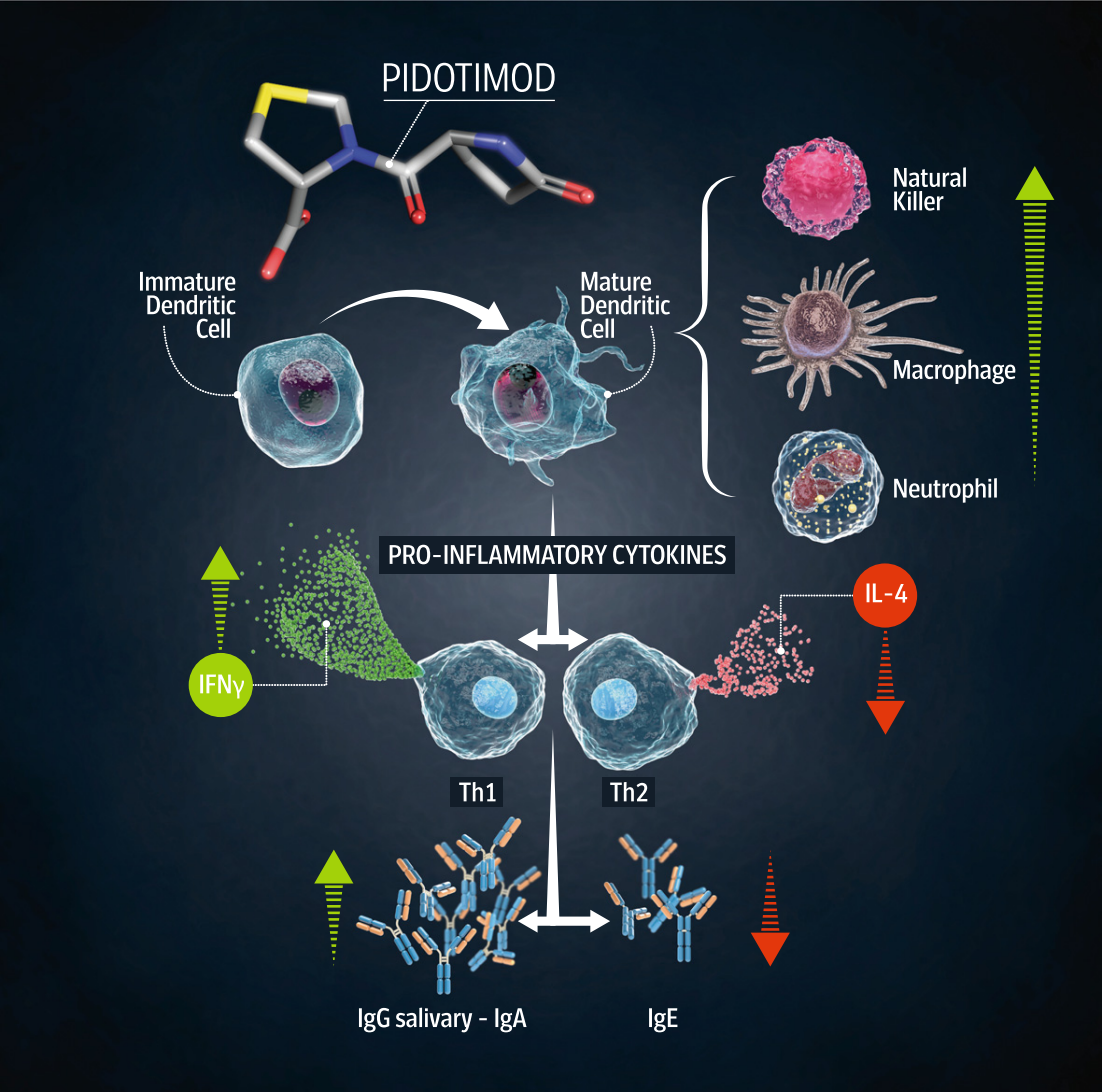
**Pidotimod: mechanism of action.**

Recent studies remark the immunomodulating role of Pidotimod: this molecule is able to improve the response to inflammatory stimuli acting on different immunological pathways (Figure 1).

Airway epithelial cells and mucosal immune cells have a crucial role against viral lower respiratory tract infections, controlling the virus and the development of inflammation and disease through innate responses, mucosal IgA responses and proinflammatory cytokines, chemokines and growth factors [4]. The “mucosal associated lymphoid tissue” (MALT) is a highly compartimentalized immunological system in which immune responses are governed by the nature of pathogen, the type of antigen-presenting cells involved (such as dendritic cells) and the local environment. It has been demonstrated that Pidotimod is able to induce DCs maturation and releasing of cytokines, to up-regulate the expression of HLA-DR and different co-stimulatory molecules [5]. An analysis of the antigen-specific salivary IgA levels has been conducted in patients (both adults and children) with frequent upper respiratory tract infections (due to Klebsiella pneumoniae, Staphylococcus aureus, Streptococcus pyogenes, Moraxella catarrhalis, Haemophilus influenzae and Streptococcus pneumoniae). Salivary fluid was collected and the presence of microorganism-specific IgA antibodies (directed against surface antigens of bacteria) was evaluated. The starting point of the study is the hypothesis that recurrent respiratory infections could be associated with secretory IgA deficiencies. The results show that both in adult and young population there wasn’t a good immune response, with low antibody titres and a very small number of patients with a strong IgA-mediated immune response. In all cases, immune response was directed only to one or two microbes. After treatment with an immunostimulant (a polyvalent bacterial mechanical lysate in this case) a significative number of patients experienced an enhancement of the concentration of salivary IgA directed against the bacteria considerated in the study (with a predominant role of IgA against Klebsiella pneumoniae) [6].

Finally, a research on microbiota reveals, through a study on TLR-7 role in respiratory influenza A virus infection in animals under antibiotic/probiotic treatment, the importance of a well-balanced intestinal flora in regulating immunity in respiratory mucosa through the up-regulation of the TLR-7 signaling pathway [7].

An Italian research demonstrates how Pidotimod can modulate airway epithelial cells functions, which play a primary defensive role both providing a physical barrier and being involved in innate and adaptive immune responses; Pidotimod, in fact, is able to upregulate the expression of toll-like receptor-2 (TLR-2) on airway epithelial cells, beside important effects on intercellular adhesion molecule (ICAM-1), IL-8 release and others. TLR plays a key role in the innate immune system: it’s a type of pattern recognition receptor and recognize molecules broadly shared by pathogenes but distinguishable from host molecules, collectively referred to pathogen-associated molecular patterns (PAMPs) [8]. Furthermore, it has been demonstrated that Pidotimod can also modulate the inflammatory cascade triggered by TLR ligands through the up-regulation of the nucleotide binding and oligomerization domain receptor (NOD-like receptor) NLRP12 [9].

### Pidotimod in children

Up to now, clinical studies on Pidotimod have been mainly conducted on children, focused on prevention of respiratory tract infections. The results show benefits from that therapy in many aspects of respiratory tract infections: a reduction of the number of infections, less day of fever, a lesser severity of signs and symptoms, and consequently a reduction in use of antibiotics and symptomatic drugs. Furthermore, the trials suggest that Pidotimod is a simple, well-tolerated and safe approach to reduce risks and symptoms correlated to respiratory tract infections [1] (Table 1).

**Table 1:** **Pidotimod: clinical trials in children**

Prophylaxis with the novel immunomodulator Pidotimod reduces the frequency and severity of upper respiratory tract infections in children with Down’s Syndrome	26 children with Down’s Syndrome	↓ nasal secretions,	
		↓ nasal obstruction	
	• M/F = 12/12	↓ mucosal hyperemia	
	• From 3 to 13 years	↓ less recurrent inflammatory episodes	
		↓ days of fever and,	
		↓ in the use of antibiotics and antipyretics.	
Immunomodulating activity of Pidotimod in children with Down Syndrome.	18 children	↑ activity of vaccine when co-administration	
	• M/F = 14/4		
	• From 3 to 10 years		
Changes of immune function in children with refractory mycoplasma pneumoniae pneumonia and effects of Pidotimod	66 children with Mycoplasma pneumoniae pneumonia	↓ of number of recurrence	↑ the number of T cells
		↓ duration of symptoms	↑ the number
		↓ duration of antibiotics treatment	natural killer cells
Comparison of effects of Pidotimod and spleen aminopeptide on clinical symptoms and Th1/Th2 cytokines in children with RRI	86 children with recurrent respiratory infection	↓duration of symptoms	↓levels of inteleukin-4 ↑levels of INF γ
	• M/F = 44/42	↓ day of treatment	
	• Mean age control group 2.9 years		
	• Mean age treatment group 3.2 years		
Observation of impact and efficacy of Pidotimod on serum interleukin-4 and gamma-interferon levels in children with asthm	52 children		↓levels of inteleukin-4 ↑levels of INF γ
	• Treatment group M/F = 20/16 mean age 9.2 years		
	• Control group M/F = 18/18 mean age 8.7 years		
The effect of Pidotimod in the prevention and Treatment of Pediatric Bronchial Asthma	100 children		↑ phagocytic activity
	• treatment group M/F = 29/21 mean from 2 to 9 years		↑chemotaxis of macrophages and neutrophilis,
	• Control group M/F = 26/24 from 2 to 10 years		↑NK cells ↑proliferation
			of CD4+
The effect of Pidotimod on serum IL-4, IFN-gamma and IgE in asthmatic children	80 children		↓level of IL-4,
	• Treatment group M/F = 24/16 from 6 to 9 years		↓IgE level
			↑level of IFN-gamma
	• Control group M/F = 23/17 from 6 to 9 years		
Immune modulator Pidotimod decreases the in vitro expression of CD30 in peripheral blood mononuclear cells of atopic asthmatic and normal children.	22 children		down-regulate CD30+
	• M/F = 14/8		
	• mean age 6.2 years		
Pidotimid may prevent recurrent respiratory infections in children	100 children	↓ the symptoms of upper and lower respiratory tract,	
	• M/F = 49/51	↓ the use of medications,	
		↓ loss of school days	
	• Mean age 4.9 years	↓ pediatric visits for RRI	

A study proposed in 2012 by Zhou and Dai compares responses of children with recurrent respiratory infections (RRI) to treatment with Pidotimod or spleen aminopeptide (a cell immunity enhancer made of peptides and nucleotides extracted from spleen of healthy animals), investigating their effect on Th1/Th2 cytokines balance. In fact, according to recent study in immunology, a functional disorder of Th1/Th2 cells, and more generally an immune hypofunction, could be strictly related to development of RRI in children. Children, divided into two randomized groups, received “Pidotimod oral solution” or “Spleen aminopeptide oral solution” in addition to routine treatment. The outcomes were clinical evolution of mycoplasmal pneumonia and the levels of inteleukin-4 and interferon-γ before and after treatment. It’s appropriate to remember that TH1 cells, which mainly mediates cell immunity, inflammatory reactions and delayed hypersensivity, are dependent on IFN-γ, while Th2 cells, involved in humoral immunity, works with dependence on IL-4. Pidotimod was able to down-regulate secrection of IL-4 and to enhance secretion of IFN-γ and other Th1 cytokines such as IL-12, thereby modulating Th1/Th2 cytokines balance; spleen aminopeptide too was able to regulates the levels of each T-cell subgroup and to enhance anti-infection ability but the study shows a statistically significant better functioning of Pidotimod [10].

A recent Italian study conducted on 100 children with at least six recurrents of respiratory tract infection in a year and at least three involving lower airways confirms that the administration of Pidotimod is able to reduce the symptoms of upper and lower respiratory tract, the use of medications, the loss of school days and pediatric visits for RRI [11].

Another significant trial has been conducted on children with Down’s Syndrome, which frequently experience acute respiratory tract infections (ARTIs) because of the associated immune defects of both specific and non-specific immunity. All children received a single dose of virosomal-adjuvanted influenza vaccine (Flu). Flu-specific IgG1 and IgG3 levels in plasma samples were determined pre- and post-vaccination, observing a significative upregulation of genes involved in activation of innate immune system and in antimicrobial activity in the group treated with Pidotimod [3].

Another study conducted on children with Down’s Syndrome shows Pidotimod’s effects from a clinical point of view. Twenty-six children with Down’s Syndrome were divided in two groups: the former (group A) received a treatment with Pidotimod and the latter (control group) was untreated. The follow-up showed that the clinical conditions were significantly improved in “group A”, in particular nasal secretions, nasal obstruction and mucosal hyperemia decreased. Parents clinical diaries too showed less recurrent inflammatory episodes and days of fever and, moreover, a cut in the use of antibiotics and antipyretics [12].

Pidotimod has been also studied in lower respiratory tract infections in children. A study conducted in 2002 inquired children with refractory Mycoplasma Pneumoniae pneumonia (MMP). The children were divided into three groups: 66 patients with refractory pneumonia, 40 with common MMP and 18 healthy controls. The first group was further divided into two parts: one received a treatment with oral azythromicyn plus pidotimod, while to the other set was administered only the antibiotic. In children with refractory MPP levels of CD3+, CD16+, CD56+ and CD4+/CD8+ ratio were lower than in common MPP and control groups. The results demonstrated that Pidotimod increases the number of T cells and natural killer cells and in clinical practice, during a follow up of six months, the study showed a decrease of number of recurrence and of the duration of symptoms and antibiotics treatment, in the group treated with Pidotimod [13].

Finally, we must remember that patients with atopic asthma are more susceptible to viral infections of respiratory tract. About this issue, an interesting study has been conducted on 22 children with mild atopic asthma and a control group of 9 children with negative history of asthma. The study investigated Pidotimod’s effect on peripheral blood mononuclear cells. Pidotimod was able to down-regulate the expression of CD30+ cells in both asthmatic and normal children. Considering of the association of CD30 with Th2-cells, this study supports the possibility that Pidotimod could affect the Th1/Th2 balance in atopic asthma with a possible additional “antiallergic” activity of the drug [14].

Pidotimod’s efficacy has been tested not only in children with respiratory tract’s pathologies but also in children with other immunity disorders, such as Henoch-Schonlein Purpura (HSP), with positive results. A study demonstrates that Pidotimod is effective for prevention of relapse of HSP thanks to an accelerated recovery of HSP immune abnormality, that consists in high serum concentration of IgA and low levels of CD3, CD4 and low CD4/CD8 ratio [15].

Pidotimod can also regulate the immunity functions in paediatric patients with nephrotic syndrome (NS), preventing the relapse rate of the disease as seen in a study conducted in 2009. The study observed children with NS for twelve months, revealing a lower relapse rate in treated patients treated than in the control group. Moreover, during treatment the CD4+ T cells increased significantly in children who received Pidotimod, with a concomitant decrease of CD8+ and a normalization of CD4+/CD8+ rate [16].

As said before, Pidotimod seems to have a beneficial effect and a good tolerability in children with pathologies due to immunological disfunctions, but further studies and larger sample size are still needed because the heterogeneity of the trials (in terms of sample size, age of subject recruited, confounding factors, duration of the intervention and misused statistical tests) is high and the possibility of publication bias can’t be escluded [3].

### Pidotimod in adults

It’s possible to find studies of Pidotimod also in adult patients with persistent or recurrent upper respiratory tract infections.

In 1995 the equipe of Prof. Zardo (Policlinico Umberto I, Rome) selected a group of fourty patients, adults and children, with recurrent pharingotonsillitis (at least five episodes with fever) in the last year. The treatment group received Pidotimod for sixty days while the control group received lyophilized polybacterial (LPV). After ninety days the patients in the first group showed a significantly increase of immune-response on Multitest (which consists in intradermical inoculation of seven different antigens and observation of cutaneous response); it means that the patients treated with Pidotimod showed an increase of number and dimension of positive cutaneous response. The groups were also divided into two further categories: patients with or without recurrent of upper respiratory tract infection after three months of observation. The 65% of people who received Pidotimod hadn’t recurrent versus the 25% of the patients who received LPV [17].

In 2004 a study considered the use of Pidotimod in senile pneumonia. One set of patients received antibiotics, expectorants and Pidotimod, the other group received only the classic treatment. The results showed a significantly different outcome between the two groups, with a better clinical progress and immunological response in treatment group, while there wasn’t a significative difference in the number of patients with or without bacterial eradication between the two groups [18].

Pidotimod’s efficacy in elder people has been also studied after “ex vivo” administration in healthy subjects. An Italian study confirms that Pidotimod is able to improve proliferation of T-cells in a group of elderly patients (selected as a model of immunodeficiency because of the changes of the immune function that usually happen in aged people), as seen before in previous study conducted on model of age-associated immunodeficiency [19].

Effect of Pidotimod has been proven not only in respiratory tract infections affected people but also in patients with genital and urological infections, such as recurrent genital herpes, non-gonococcal urethritis and serofast syphilis [20-23].

Also in Human Papilloma Virus (HPV) genital infection, which causes vulvar papillomatosis, a treatment with Pidotimod is able to lead to a regression of the papillomatous lesions, with better results if compared with a control group treated with placebo. Furthermore, the efficacy of Pidotimod against HPV genital lesions seems comparable to that of α- and β-interferon but with a better safety profile [24]. Immunosuppression is, in fact, a risk factor for HPV infections and related pathological conditions and it’s clear the importance of a complementary immunostimulatory treatment, beside cryotherapy or laser therapy, to reduce the risk of recurrences. It has been also demonstrated that the combination of Pidotimod and vitamin C has a certain efficacy against female genital warts [25].

In literature it’s possible to find a study on the effects of Pidotimod on immune function in patients with chronic hepatitis C. In these patients the diagnosis of chronic hepatitis C is in according to the diagnostic criteria revised in the “Fifth National Conference on Contagious and Parasitic Disease”. The study compares two groups, twelve cases of healthy individual and fourty-six patients with chronic hepatitis; Pidotimod is administered 800 mg once a day for 1-2 months. The results show that IL-2, IL-4 and IL-6 increased significantly in the treatment group, but there is no evidence that Pidotimod can improve the expression of IFN-γ in both groups; moreover, the ratio of Th1/Th2 decreased in treatment group. In summary, the study demonstrated that Pidotimod is able to improve the expression of Th1 cytokines and increases the cellular immune function and viral clearance ability of the host [26] (Table 2).

**Table 2:** **Pidotimod: clinical trials in adults**

**Study**	**Population**	**Clinical outcome**	**Laboratory outcome**
Evaluation of the efficacy of Pidotimod in the exacerbations in patients affected with chronic Bronchities	181 COPD	↓Frequency of relapses	Expectoration characteristics
	• M/F = 117/64	↓ to time of relapses,	↑ spirometric parameters
	Mean age 62.5 years	↓ time of recovery	
Pidotimod activity against chronic bronchitis exacerbation	580 COPD	↓ fewer infectious exacerbations	
	• Treatment group M/F = 94/31 mean age 66.2 years	↑ better response to antibiotics treatment	
		↓days out of work	
	• Placebo treatment M/F = 200/63 mean age 65.2 years		
Immunological function of T-lynphocyte in the elderly with chronic obstuctive pulmonary disease during acute exacerbations medication intervention	103 COPD	↓day of hospitalization	↑ the cell immune response in elderly patients
	• Elderly COPD intervention group M/F = 23/9mean age 7.1 years	↓ cost of hospitalization	
			↑ spirometric parameters
	• Elderly COPD control group M/F = 22/12 mean age 69.9 years		
	• Non elderly COPD control group M/F = 15/10 mean age 56.2 years		
Ex vivo evaluation of Pidotimod activity in patients with chronic obstructive pulmonary disease.	52 COPD		↑T-cells activity ↑stimulate macrophages and granulocytes
	• Treatment group		
	M/F = 13/13 mean age 65.92		
	• Control group M/F = 10/16 mean age 62.46		
Treatment of 30 cases of senile pneumonia with Pidotimod.	62 elderly with pneumonia	↓n° of recovery	↑ neutrophilic bactericidal
		↑ bacterial eradication	
			↑ IgG level
	• M/F = 33/29		
	• Mean age 72 years		
Valutazione dell’attività del Pidotimod sulla secrezione di IgA in pazienti affetti da BPCO	20 COPD		↑of secretory IgA in the sputum,
	• From 40to 75 years		
			a stabilization of the level.
Valutazione sperimentale controllata dell’attività di Pidotimod in paziente affetti da BPCO	85 COPD	↓ number of exacerbations	
	• M/F = 54/31		
Pidotimod activity against chronic bronchitis exacerbations	580 COPD	↓number of infectious exacerbations	
	• Treatment group M/F 94/31 mean age 66.2 years	↓duration of Exacerbations	
		↓duration of therapy	
	• Control group		
	M/F = 200/63 mean age 62.46 years		
Naturally occurring immune response against bacteria commonly involved in upper respiratory tract infections: analysis of the antigen-specific salivary IgA levels.	21patients		↑of the concentration of salivary IgA
	• M/F = 12/10		
	• mean age 41 years		
Treatment of 30	62 patients	↓ number of hospitalization	↑ neutrophilic activity
cases of senile pneumonia with Pidotimod	• M/F = 33/29		↑level of IgG
	• Mean age 72 years		
Pidotimod nel trattamento delle faringotonsilliti recidivanti	40 adults + children with recurrent pharingotonsillitis	↓ recurrent of upper respiratory	↑ of immune-response on Multitest
	• M/F = 13/21		
	• Mean age 23.2 years		

### Pidotimod in asthma

Asthma is a common chronic inflammatory disease of the airways characterized by variable and recurring symptoms, reversible airflow obstruction and bronchospasm. Common symptoms include wheezing, coughing, chest tightness, and shortness of breath. Asthma is thought to be caused by a combination of genetic and environmental factors. It is clinically classified according to the frequency of symptoms, forced expiratory volume in one second (FEV1), and peak expiratory flow rate. Asthma may also be classified as atopic (extrinsic) or non-atopic (intrinsic) where atopy refers to a predisposition toward developing type 1 hypersensitivity reactions.

Thanks to many studies conducted in the last years is now clear that there is a relationship between the exposure to viral pathogens and the development of allergic diseases but in this context viruses seem to be “a double-edged sword”, as affirmed in the past. In fact, while environmental exposure to some viruses has been associated with an increased resistance to allergic diseases, on the other hand we can observe that severe respiratory viral infections during childhood increase the risk of a subsequent development of asthma [27].

In regard to that, we should remember the “hygiene hypothesis”, which states that a lack of early childhood exposure to infectious agents, symbiotic microorganisms (e.g. gut flora or probiotics), and parasites increases susceptibility to allergic diseases by suppressing the natural development of the immune system. In particular, the lack of exposure is thought to lead to defects in the establishment of immune tolerance.

We can affirm that the pathogenesis of asthma is the result of many combined factor, in particular Th1/Th2 cytokine imbalance, IgE-dependent immediate type hypersensitivity reactions and eosinophilic infiltration mediated by overactive Th2 cytokine responses. Interleukine-4 (IL-4) inhibits production of interferon-γ (IFN-γ), promotes local airways inflammation and development and progression of asthma; IFN-γ, on the other hand, has an opposite role, protecting from these inflammatory mechanisms which lead to asthma. Therefore, correcting the cytokine imbalance by regulating IL-4 and IFN-γ seems to be a promising approach to the treatment of asthma in children [28].

Clinically, asthma can be stable for weeks or even months but acute exacerbations can be caused by many triggering agents: among others, both viral and bacterial infections of the upper respiratory tract can lead to a worsening of the disease.

It’s now clear the importance of prevention of chronic lung diseases, like asthma. According to the “NHLBI Workshop on the Primary Prevention of Chronic Lung Disease”, there are three kind of intervention to be tested in clinical trial now or in the future: preventing asthma through prophilaxis against respiratory syncytial viruses and human rhinovirus infections of the airways; immune modulation, using prebiotics, probiotics and bacterial lysates; and prevention of allergen sensitization and allergic inflammation using anti-IgE [29].

A study conducted by Zhai in 2011 shows the immunomodulator effect of Pidotimod in prevention and treatment of pediatric bronchial asthma. In that study the frequency, degree and duration of asthmatic attack were observed after treatment and markers of immunological response were measured before and after treatment. Asthma is closely associated with CD4+/CD8+ imbalance and the use of CD4+ cells as a target point of immune regulation in asthma have been proposed. The study shows that Pidotimod can increase the phagocytic activity and chemotaxis of macrophages and neutrophilis, activate natural killer cells and promote mitosis-induced proliferation of lymphocites in order to restore normal values of low CD4+ and a normal CD4+/CD8+ ratio. It’s important to underline that during the clinical observation period none of the children experienced a serious adverse drug reaction, indicating that the drug has good safety [30].

Other similar studies exist and confirm the role of Pidotimod on IL-4 and IFN-γ in children with asthma. A clinical trial conducted by Ma and Sun in 2011, besides confirming the efficacy of Pidotimod against cytokines imbalance, shows its action on IgE level, which decreases significantly after the treatment. In addition to this important outcome, the study shows an improvement in pulmonary function data, such as forced expiratory volume in 1 second (FEV1) and peak expiratory flow rate (PEF) in treatment group if compared with control group [31].

### Pidotimod in COPD

Chronic obstructive pulmonary disease (COPD) is one of the main causes of morbidity, hospital admission and loss of working day in industrialized countries. COPD is a type of obstructive lung disease characterized by chronically poor airflow with not reversible obstruction at spirometry. Its clinical features include shortness of breath, cough, sputum production and the typical flares, characterized by a worsening of breathing difficulties and cough and purulent sputum. These exacerbations are often due to respiratory tract infections.

Pidotimod has been tested in patients with an infectious relapse of COPD in a double-blind randomized trial and the results indicate that it’s significantly more effective than placebo, in relation to time and frequency of relapses, as well as expectoration characteristics, time of recovery, spirometric parameters and laboratory tests. According to literature, in fact, Pidotimod stimulates cell-mediated immunity and the study confirms the results obtained by many authors, who affirmed that Pidotimod acts on cell-mediated immunity increasing mitogen-induced lynfocites blastogenesis, potentiates rosette activity, activates macrophages potentiating chemotaxis and superoxide anion production and protects against experimentally induced infections [32].

Anyway, this is not the only study which investigates the role of Pidotimod in exacerbations of chronic bronchitis. In particular, some Italian trials focus on this issue, comparing Pidotimod-treated patients with control patients (treated with placebo). The results show an enhancement of clinical (in particular fewer infectious exacerbations and better response during acute infections) and immunological parameters both during the treatment and during the follow-up period, suggesting a protective action of the drug and the possibility of the use of Pidotimod in cyclic therapy [33]. Pidotimod in fact is able to potentiate T-cells activity not only during treatment but also after the end of therapy (till five weeks) and to stimulate macrophages and granulocytes, as demonstrated in other trials [34].

In literature we can also find a study in elderly with chronic obstructive pulmonary disease with COPD exacerbations. The study investigated the role of Pidotimod demonstrating that it’s able to improve the cell immune response in elderly patients with COPD flares who have a suppressed T-cell immune response if compared with patients of the same age in the healthy group and with those in the non-elderly COPD control group. The causes of the initial abnormality of T-cells in patients with COPD can be ascribed to malnutrition, long-term smoking, repeated bacterial or viral infections, glucocorticoids therapy and severe hypoxemia [35].

The exacerbations of COPD are proportional to the severity of disease (which is strictly related to FEV1 decline). An Italian study evaluated eighty-five patients with severe COPD (GOLD 3 stage) divided into two groups: the former received a vaccine against influenza virus plus Pidotimod, the latter received only the vaccine for 4 months. The outcomes of the study were the number of patients with exacerbation and the number of exacerbations for each patient. The results show a significant difference between the two groups of patients in terms of number of exacerbations, with a reduction of exacerbations in treatment group. Instead, there was no significant difference in the duration of the symptoms of exacerbation between the two groups. Furthermore, Pidotimod represents a more safe solution for patients subjected to vaccination who need an immunostimulant treatment, in particular if compared to lyophilized polibacterial, which can’t be administered for 30 days before vaccination [36].

Focusing on bacterial exacerbations, we must remember a study conducted on patients divided in two groups and treated with Pidotimod plus Augmentin or placebo plus Augmentin. In this case too, the efficacy of Pidotimod has been shown by a faster remission of symptoms and a higher number of positive responses to skin test (testing immunocompetence) in the former group. Furthermore, the safety of the drug has been confirmed one more time, with a very low incidence of side effects [37].

An old study shows that the use of Pidotimod, if compared with placebo, increases the secretory IgA in patients with COPD; in particular twenty patients were selected and were treated with the drug for 4 weeks and observed during a follow-up period of 2 weeks. The level of secretory IgA was assessed before, during and after the treatment, and also the viscosity and the elasticity of sputum was assessed. The result showed that the patients treated with Pidotimod had a significant increase of secretory IgA in the sputum, and also a stabilization of the level. During the follow up was observed a linearity inclination to the physiological value of secretory IgA. In conclusion Pidotimod, during the stabilization phase, supports a good protection to the infection because IgA activate the complement system and decrease the bacterial bond to the mucosal surface. On the other hand, data related to viscosity and linearity of sputum didn’t change significantly between the two groups [38].

### Pharmacology

Pidotimod has been investigated in different pharmacokinetic studies in healthy volunteers.

The studies show that plasma levels of Pidotimod after parenteral administration follow a second order pharmacokinetic while after oral administration follow a first order pharmacokinetic. The molecule is early absorbed and its half-life is approximately four hours. The oral bio-availability is 42-44%; the compound is eliminated unchanged through urines without being metabolized. Plasma clearance is 5 l × hE-1, and the apparent distribution volume is 30 liters, so it is not shown accumulation neither auto-induction phenomena. It has also been demonstrated that the different oral formulations (tablets, sachets and vials) are bioequivalent [39].

A study conducted on mice investigates the use of Pidotimod in “water-in-oil-in-water” (W/O/W) double emulsion (hydrophilic bioactive substances stored into droplets encapsulated by an oil coating) as a delivery system with an enhanced oral bioavailability, higher than Pidotimod control solution. The results of the study confirm the initial hypothesis and, moreover, suggests an extra absorption pathway for W/O/W emulsion because the plasma concentration peaking time is significantly prolonged. Furthermore, the plasma half-life is prolonged and the elimination is slowed. These results suggest a different absorption mechanisms of W/O/W, which may be absorbed into lymphatic circulation through the cycle role of bile [40].

In the end, we can affirm that Pidotimod has a good safety because it has shown a very good safety in pretty much all the studies, with a low risk of collateral effects and, moreover, we should remember a study conducted on mice which shows that Pidotimod has not mutagenic potential [41].

## Conclusion

Despite the use of antibiotics and vaccines, the frequency of respiratory tract infections is still high and these infections interest a wide range of patients, from children to aged people, including in particular these extreme categories because of the deficiency of their immune system, due to immaturity in the former case and to “immunosenescence” in the latter. For that reason immunostimulant drugs are getting more important to prevent and to attenuate infections.

Pidotimod (3-L-pyroglutamyl-L-thiazolidine-4carboxylic acid) is a synthetic dipeptide with immunomodulatory properties. Studies conducted in vivo on different populations and mice and also in vitro studies show its efficacy and safety. The outcomes considered are both clinical and laboratory parameters.

In vivo studies have been conducted on:

- children with and without lower or upper respiratory tract infections (also recurrent)
- a restricted population of children with Down’s Syndrome which are often affected by acute respiratory tract infections
- children and adults with asthma, in which respiratory tract infections can lead to a worsening of the disease
- children with immunological disorders, such as Henoch-Schonlein Purpura, or with nephrotic syndrome
- adults with persistent or recurrent upper and lower respiratory tract infections (such as pneumonia and pharyngotonsillitis) and healthy adults
- adults with genital or urological infections (in particular HPV) or affected by chronics hepatitis C
- adults with COPD exacerbations

The common end-point of these studies is that Pidotimod has an immunomodulatory activity which is able both to improve the clinical conditions of patients and to enhance and stimulate their immunity cells (lymphocytes but not only) functions acting on adaptive and innate immunity. Pidotimod is also able to increase the concentration of salivary IgA directed against bacteria; furthermore, it can modulate airway epithelial cells functions up-regulating the expression of toll-like receptors and acting on adhesion molecules. Studies remark that also the balance of intestinal flora can influence the immunity in respiratory mucosa, remembering the role of probiotics and immunostimulants.

According to studies conducted on patients with atopic asthma, it seems that Pidotimod could affect T-lymphocytes balance with a possible addictional anti-allergic activity. Furthermore, it has been demonstrated an improvement of FEV1 and PEF in asthmatic patients treated with Pidotimod.

Main clinical outcomes are the reduction of the number of infectious episodes, lesser severity of signs and symptoms and, consequently, a reduction in use of antibiotics and symptomatic drugs, less working and school days lost, less mortality and morbidity.

The studies considered give positive results, confirming Pidotimod’s efficacy, even when compared to other immunostimulants or combined with antibiotic therapy. Furthermore, many studies show a good safety profile of the drug, without recording serious adverse events and mutagenic potential, and a very low incidence of side effects. Pidotimod is also a more safe solution in patients subjected to vaccination, if compared to lyophilized polibacterial, which can’t be administered for thirty days before vaccination.

Finally, we must remember that pharmacokinetic studies demonstrated that different oral formulations (such as tablets, sachets and vials) are bioequivalent.
